# A retrospective study of external beam radiation, neutron brachytherapy, and concurrent chemotherapy for patients with localized advanced carcinoma of the esophagus

**DOI:** 10.1186/s13014-014-0294-3

**Published:** 2014-12-17

**Authors:** Kai Ma, Qifeng Wang, Tao Li, Huiming Liu, Bo Liu, Xitang Jia, Shufeng Li, Junyuan Lang, Mingzhi Zhang

**Affiliations:** Department of Oncology, The First Affiliated Hospital of Zhengzhou University, Zhengzhou, Henan 450052 China; Department of Radiation Oncology, Sichuan Cancer Hospital, Chengdu, China; Department of Radiation Oncology, Changzhi Cancer Hospital, Changzhi, 046000 People’s Republic of China; General Hospital of Jincheng Anthracite Mining Group Co.Ltd, Jincheng, China

**Keywords:** Esophageal cancer, Californium-252, Neutron brachytherapy, Chemotherapy, Late complication

## Abstract

**Purpose:**

The aim of this study was to retrospectively observe and analyze the long-term treatment outcomes of a total of 140 esophageal cancer patients who were treated with californium-252 (^252^Cf) neutron brachytherapy (NBT) in combination with external beam radiotherapy (EBRT) and concurrent chemotherapy (CCT).

**Methods and materials:**

From January 2002 to November 2012, 140 patients with esophageal cancer underwent NBT in combination with EBRT and CCT. The distribution of the patient numbers over the various cancer stages of IIA, IIB, and III were 43, 7, and 90, respectively. The total radiation dose to the reference point via NBT was 8–25 Gy-eq in two to five fractions with one fraction per week. The total dose via EBRT was 50–60 Gy, which was delivered over a period of five to six weeks with normal fractionation. Fifty-four and 86 patients received tegafur suppositories (TS) and continuous infusion of fluorouracil (5-Fu) with cisplatin (CDDP), respectively.

**Results:**

The median follow-up time was 42 months. The minimum follow-up was three months, and the maximum was 106 months. The overall median survival including death from all causes was 29.5 months. The five-year overall survival rate (OS) and local control (LC) were 33.4% and 55.9%, respectively. The chemotherapy regimen was a factor that was significantly associated with OS (p = 0.025) according to univariate analysis. The five-year OSs were 27.4% and 44.3% for the PF and TS chemotherapy regimens, respectively. Regarding acute toxicity, no incidences of fistula or massive bleeding were observed during this treatment period. The incidence of severe, late complications was related to the PF chemotherapy regimen (p = 0.080).

**Conclusions:**

The clinical data indicated that NBT in combination with EBRT and CRT produced favorable local control and long-term survival rates for patients with esophageal cancer and that the side effects were tolerable. A reasonable CRT regimen can decrease the rate of severe, late complications.

## Introduction

In 2013, an estimated 17,990 cases of esophageal cancer were diagnosed in the United States, and approximately 15,210 people died from this disease [1]. Worldwide, an estimated 482,000 new esophageal cancer cases were diagnosed, and approximately 407,000 deaths occurred in 2008 [2]. Although surgery continues to be the standard approach for the majority of localized esophageal cancers, the cure rates following surgery alone are poor with three- to five-year survival rates that range from 6% to 35% [3-5]. The management of loco-regional or locally advanced esophageal cancer has shifted from single modality surgery or radiation approaches to trimodal approaches involving the addition of chemotherapy. The current trimodal approach, which combines chemotherapy, radiation therapy, and surgery, has significantly improved prognoses, and several studies have shown improved survival rates [6]. However, many patients cannot tolerate or decline surgery; for such individuals, definitive concurrent chemoradiotherapy (CCRT) is the standard approach.

A radiation therapy oncology group study (RTOG 8501) demonstrated a survival benefit of the addition of platinum-based chemotherapy to radiation compared to radiation alone for patients with nonsurgical esophageal cancer [7,8]. Despite improved local, regional, and distant control and increased survival, approximately 50% of patients will have persistent local disease or recurrence [7-9]. To enhance the local control rate for advanced cancer, a combined treatment with CRT and a brachytherapy boost seemed promising. There were a few published results of external beam radiation (EBRT), brachytherapy boosts, and concurrent chemotherapy for meaningful numbers of patients. However, a high incidence of treatment-related esophageal fistulas was observed in the RTOG 92–07 [10]. We present the results of a retrospective analysis of a large consecutive series of patients with locally advanced esophageal cancer who were treated with a multimodal strategy. The main objective was to assess the overall survival and local control rates after combined chemotherapy and irradiation (EBRT plus neutron brachytherapy). We also evaluated the treatment tolerance, prognostic factors and patterns of failure.

## Materials and methods

### Patients’ characteristics

From January 2002 to November 2012 at the Changzhi Cancer Hospital, a total of 140 consecutive patients with localized esophageal cancer were referred to our department for EBRT and ^252^Cf neutron brachytherapy (NBT) and concurrent chemotherapy. Before entry into the study, all patients underwent a barium swallow and upper gastrointestinal endoscopy, chest CT scans, B-type ultrasonography of the neck and abdomen, collection of blood parameters (including hematology), and biochemical investigations (including liver function tests). The selection criteria included a Karnofsky performance score ≥80, tumors ≤10 cm in length on endoscopy and/or barium swallow, the capability to take semifluid food, no hoarseness of the voice, no active bleeding, no perforation of the esophagus, no remote metastasis, and no prior malignancy. The cancers in the lower esophageal sphincter were all identified near the opening of the sphincter, and no clear invasion into the stomach was observed. The cancer stages (T/N/M) were determined based on the CT and endoscopy results. All of the patients provided informed consent before treatment, which was in accordance with the Declaration of Helsinki, and this study was also approved by the Ethics Committee of Changzhi Cancer hospital. The demographic data and tumor characteristics of each group are given in Table 1.

**Table 1:** **Patient and tumor characteristics**

**Characteristics**	**Total (%)**	**TS**	**PF**	**p value**
Gender				0.395
Male	84(60.0)	30(55.6)	54(62.8)	
Female	56(40.0)	24(44.4)	32(37.2)	
Age(years)				0.028
≤65	86(61.4)	27(50.0)	59(68.6)	
>65	54(38.6)	27(50.0)	27(31.4)	
The length				0.354
≤5 cm	58(41.4)	25(46.3)	33(38.4)	
>5 cm	82(58.6)	29(53.7)	53(61.6)	
Tumor location				0.800
Upper	33(23.6)	11(20.4)	22(25.6)	
Middle	92(65.7)	38(70.4)	54(62.8)	
Lower	15(11.7)	5(9.2)	10(11.6)	
T stage				0.026
T2	22(15.7)	13(24.1)	9(10.5)	
T3	51(36.4)	22(40.7)	29(33.7)	
T4	67(47.9)	19(35.2)	48(55.8)	
N stage				0.002
N0	68(48.6)	35(64.8)	33(38.4)	
N1	72(51.4)	19(35.2)	53(61.6)	
6th AJCC stage				0.007
Iia	43(30.7)	24(44.4)	19(22.1)	
Iib	7(5.0)	4(7.4)	3(3.5)	
III	90(64.3)	26(48.2)	64(74.4)	
RT Dose				0.809
≤66 Gy	89(63.6)	35(64.8)	54(62.8)	
≥67 Gy	51(36.4)	19(44.4)	32(37.2)	

*Abbreviations:* CRT = chemotherapy plus radiotherapy; RT = radiotherapy alone; OS = Overall survival rate; LCR = local control rate.

The characteristics of the analyzable patients were as follows: 84 men and 56 women, aged 44–70 years (median 63), and KPS scores of 90 in 58 patients, and 80 in 82 patients. The main locations of the lesions were upper thoracic in 33 patients, middle thoracic in 92, and lower thoracic in 15, and middle thoracic lesions accounted for 88.3%. The major axis of the lesion was 6.0 cm in median length with a range of 3.0 to 10 cm. In the TNM classification, the T-stage was T2 in 22, T3 in 51, and T4 in 67 patients; the N-stage was N0 in 68, and N1 in 72 patients. The responses at the time of registration (i.e., the response to an external irradiation of 50 Gy) were complete responses (CRs) by barium swallow and endoscopic findings in 98 (70%) patients, partial responses (PR) in 39 (27.9%), and no change (NC) in three patients. All patients received concurrent chemotherapy with EBRT + NBT. Fifty-four and 86 patients received TS and PF regimen chemotherapies, respectively. The characteristics of the patients differed between the TS regimen group and the PF regimen group (Table 1).

### Radiotherapy

The high-LET (^252^Cf-based) NBT and the low-LET EBRT were interchangeably implemented over the treatment time period. The EBRT was performed with a 6-MV linear accelerator. The treatment field size was determined according to the CT and barium swallow test results. The two-field technique (one anterior field and one posterior field) or the three-field technique (one anterior field and two posterior fields) was used to treat the upper segment of the esophagus. Only the three-field technique (one anterior field and two posterior fields) was used to treat the middle and lower segments of the esophagus. The upper and lower boundaries of the treatment field were determined by adding 3–5 cm from the visible disease area shown on the CT/barium swallow images. In general, the width of the treatment field was 6–7 cm, and the total length was approximately 15 cm. The EBRT followed the normal fractionation with five fractions per week, one fraction per day, and 1.8-2.0 Gy per fraction for a total of 25–30 fractions, and the total treatment time was five to six weeks. The total dose via EBRT was 50–60 Gy, which was delivered over a period of five to six weeks with normal fractionation.

**Figure 1:**
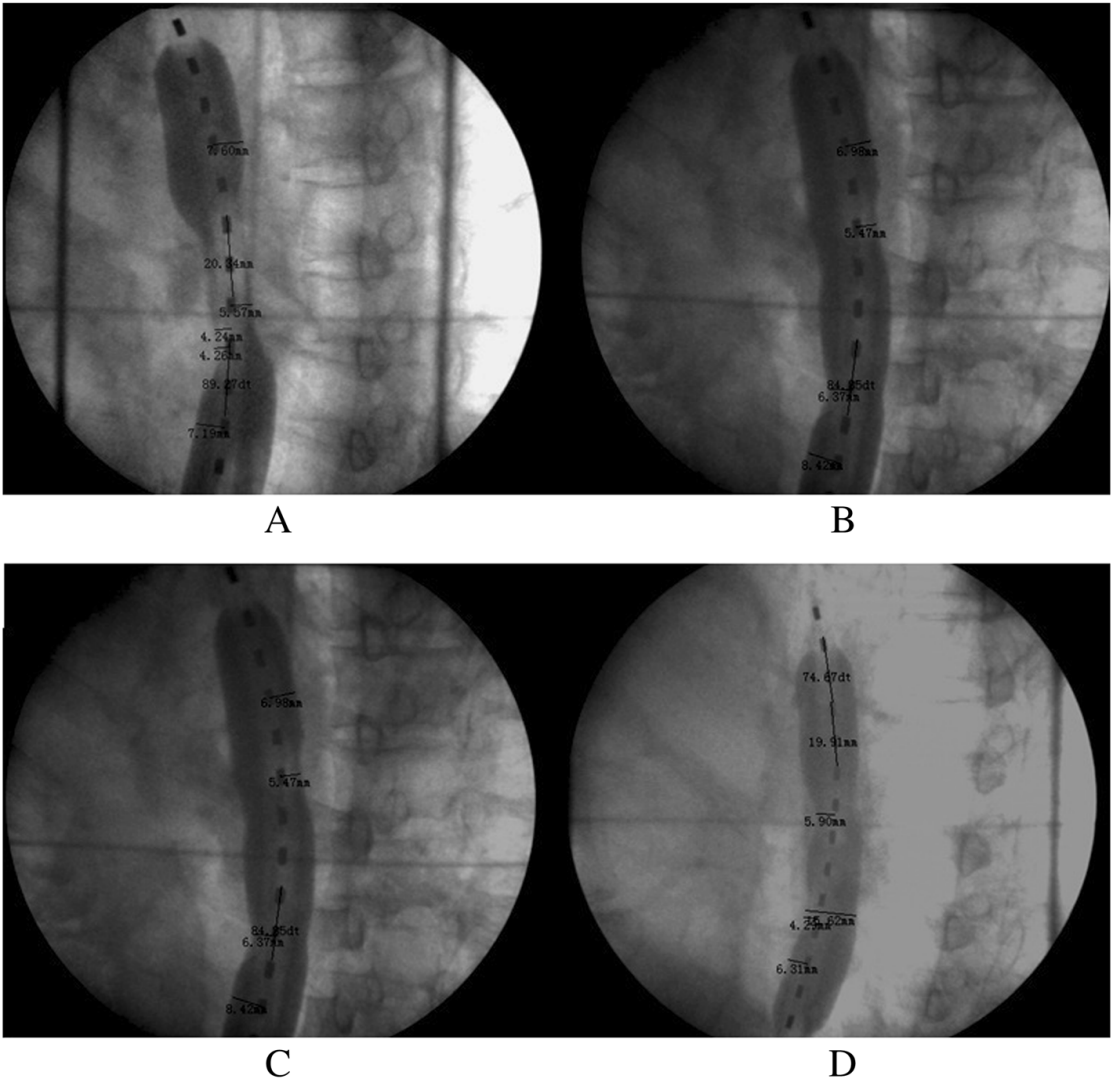
**Images (A-D) showing the tumor regression conditions before each of the four NBT treatments under an X-ray treatment-planning simulator.**

NBT with a one-balloon applicator (Figure 1) was used in conjunction with the ^252^Cf LZH-1000 remote after-loading system (Linden Science and Technology Co, Shenzhen, China). The physical characteristics of the ^252^Cf neutron, the characteristics of the applicator, and the process of NBT have been described in detail by Liu H [11,12]. The dose was prescribed to the reference point, which was located 10 mm from the center point of the source capsule in the transverse direction. Figure 1 shows an X-ray image taken while the applicator and the source were both inserted into the esophagus of a patient. In Figure 1, the water balloon can clearly be seen to be filled with an X-ray contrast agent. The dose was prescribed to the reference point, which was located 10 mm from the center point of the source capsule in the transverse direction. The total radiation doses (to the reference point) given to each patient varied between 8–25 Gy-eq in two to five fractions with 4–5 Gy-eq per fraction per week.

### Chemotherapy

Two concurrent chemotherapy regimens were used: 140 patients received concurrent chemoradiotherapy. Fifty-four patients received tegafur suppositories (TS) of 500 mg/m^2^ that were given on days 1–14 and days 21–35. Eighty-six patients received continuous infusion of fluorouracil (5-FU, 750 mg/m2) on days 1 through 5 and days 29 through 33 with cisplatin (75 mg/m^2^) given on days 1 and 29.

### Toxicity assessment and follow-up

The patients were examined weekly during the course of the external beam radiation. Weekly blood tests were obtained, and any admissions for treatment-related complications were recorded. All adverse events were graded according to the National Cancer Institute’s Common Terminology Criteria for Adverse Events, version 3.0 [13]. The patients typically underwent follow-up examinations every 3–6 months after the completion of treatment. Tumor responses and nodal diseases were evaluated with repeated CT scans, barium swallow studies, and endoscopy.

### Statistical analyses

The patients were grouped according to the different chemotherapy regimens (TS and PF). Pearson’s chi-square tests were used to assess the relationships between the frequency data. The overall survival (OS) time was calculated from the date of consultation until death or the last follow-up. Local and regional failures were defined by persistent and/or recurrent primary tumors and regional lymph nodes. The time to first failure, time to local failure, and time to any distant metastases were calculated from the date of consultation. The overall survival (OS) and local control (LC) rates were estimated using the Kaplan-Meier method. Nine factors were included for the univariate analyses of OS and LC and included gender, age, Karnofsky performance score (KPS), tumor location, tumor length, T stage, N stage, TNM stage, and radiation dose. The log-rank test was used to assess the survival differences between the groups. The data were analyzed using SPSS software (version 20.0, SPSS Inc., Chicago, IL, USA).

## Results

### Survival and local control

The median follow-up time was 42 months. The minimum follow-up was three months, and the maximum was 106 months. The overall median survival including death from all causes was 29.5 months. The three and five-year overall survival rates (OS) were 46.3% and 33.4%, respectively. The five-year local control rate for the entire group was 55.9%. The survival rates, median survivals, and local control rates (LC) according to stage are shown in Table 2. As shown in this table, a total of nine factors were considered and analyzed for their relevance to overall survival and local control. These factors included gender, age, tumor length, tumor location, chemotherapy regimen, stage T, stage N, AJCC stage, and radiation dose. Among these factors, only chemotherapy regimen was found to be relevant to overall survival (*p* = 0.025), and none of the factors were related to local control. The five-year Oss were 27.4% and 44.3% for the PF and TS chemotherapy regimen, respectively (*p* = 0.025).

**Table 2:** **Local control and overall survival according to stage and chemotherapy regimen**

**Characteristics**	**Total**	**TS**	**PF**	**p value**
Stage II	N = 50	N = 28	N = 22	
5-year LC	60.1%	64.8%	53.9%	0.526
5-year OS	35.7%	40.5%	32.4%	
Median survival	32.1 months	30.8 months	35.1 months	0.855
Stage III	N = 90	N = 26	N = 64	
5-year LC	53.3%	56.6%	49.6%	0.244
5-year OS	32.3%	47.1%	25.9%	
Median survival	24.0 months	42.4 months	14.6 months	0.015
Total	N = 140			
5-year LC	55.9%	61.2%	51.1%	0.185
5-year OS	33.4%	44.3%	27.4%	
Median survival	29.5 months	37.7 months	18.6 months	0.025

### Patterns of failure

As of the date of the last follow-up (March 31, 2013), the total number of failures (including local recurrences, remote metastasis, and deaths) was 94 (67.1%). There were 56 recurrences (40.0%), among which, 53 (37.9%) were in-field recurrences, and three (2.3%) were out-of-field recurrences. Among the three out-of-field recurrences, one occurred in the supraclavicular lymph nodes, and two occurred in the intra-abdominal lymph nodes. A total of 18 cases of remote metastases were found, among which, the number of metastases to the lungs, liver, bone and others tissues were five, three, two, and eight, respectively. A total of nine deaths as results of fistula and hematemesis were observed. One patient died of a secondary cancer. A total of 17 deaths were found to be caused by reasons other than the original cancer or the radiation treatment.

### Treatment toxicity

All 140 patients completed the planned NBT and EBRT and concurrent chemotherapies. In terms of acute toxicity, no perforations were observed during this treatment period. In total, 107 (76.4%) patients developed Grade 2 hematologic toxicities. Dysphagia was relieved after the second or third NBT treatment in 126 cases (90%), and temporary feeding tubes were not required in the majority of the patients. Esophagitis of Grade 2 or more as expressed by clinical odynophagia was observed in 100 cases (71.4%). In total, six patients had Grade ≥ 2 irradiation dermatitis. From the time of treatment completion to the development of local-regional recurrence or death at the follow-up time, four (2.9%) and five (3.6%) patients experienced fistulas and massive bleeding, respectively. As shown in Table 2, the incidence of severe, late complications was related to the PF chemotherapy regimen (8/86, 9.3%) compared to the TS regimen (1/54,1.9%; p = 0.080). In total, 75.0% of the patients resumed normal swallowing, and 3.6% (5/140) had some residual dysphagia (non-malignant) that required intermittent dilatation. The other acute toxicities and late complications were not significantly related to higher total doses or the receipt of different CCRT regimens.

## Discussion

To our knowledge, this is the first reported clinical experience of the treatment of esophageal cancers using NBT, EBRT, and concurrent chemotherapy. The safety and efficacy of this comprehensive treatment appear promising. We also found that, first, NBT + EBRT and concurrent chemotherapy is safe and beneficial in terms of local control in the radical treatment of patients with esophageal cancer, and second, the OS rate was significantly increased, and the late complication rate was significantly decreased in patients who received the TS chemotherapy regimen.

Prior to this report, the published results related to external beam radiation, brachytherapy boost, and concurrent chemotherapy in a meaningful number of patients have been few [14]. Table 3 summarizes these experiences along with our own. The present study documented no treatment-related fistulas in the patients, although direct comparison to the other reports is hampered by the differences in staging, classification, response end points, and the duration of follow-up. Differences in treatment regimens or sequencing might account for some of the observed differences in toxicity. The present study showed survival benefits from the addition of NBT to EBRT and concurrent chemotherapy in the treatment of locally advanced disease that resulted in short-term effects, and these findings are similar to those in the reports of Montravadl *et al.* [15] and Sharma *et al.* [16]. This treatment strategy resulted in better long-term survival and local control as other authors have reported [14-16]. The primary reason for these effects is that the incidence of late, severe complications was reduced significantly by this treatment strategy.

**Table 3:** **Clinical results of external beam radiation, brachytherapy boost, and concurrent chemotherapy**

**Authors (Ref.)**	**Montravadl** ***et al.*** **[** 15 **]**	**Sharma** ***et al.*** **[** 16 **]**	**RTOG9207** **[** 14 **]**	**Present study**
No. of pts.	40	100	50	140
BT Gy/fraction	10/2	15(group 1)20(group 2)/1	15/3	8-25/2-5
Interfraction interval	2 wks	1 wks	1 wks	1 wks
Applicator diameter	1 cm	NS	4-6 mm	0.9 cm
EBRT Gy/fraction	40-55 Gy/4-6 weeks	50/28	50 Gy/5 wks	50-60 Gy/5-6 wks
CT (pts)	Mito C 10 mg/m^2^ i.v. Days 1,29; 5-FU 1000 mg/m^2^/day 3 4 days	5-Fu 500 mg/m^2^, 12 h before brachytherapy	DDP 75 mg/m2 Day 1 Wk 1,5,8,11	TS) 500 mg/m2 given on days 1–14 and days 21–35, or DDP 75 mg/m2 Day 1 Wk 1,5, 5-FU 750 mg/m2/day, days 1–5, Wk 1,5
			5-FU 1000 mg/m^2^/day 3 4 days, Wk 1,5,8,11	
Fistula (%)	0%	12%	12%	2.9%
Bleeding (%)	NS	4%	NS	3.6%
Ulcer (%)	NS	29%	NS	NS
Stricture (%)	23%	16%	4%	3.6%
OS(%)	3 yrs 40%	5 yrs 8%(group 1)	3 yrs 29%	5 yrs 33.4%
		5 yrs 23%(group 2)		
LC (%)	85% Complete response; 78% Local control	NS	74% Complete response; 37% Local control	70% Complete response; 55.9% Local control

RTOG: Radiation Therapy Oncology Group; EBRT: external beam radiation therapy; HDR: high-dose-rate; NS: not specified; Adenoma: adenocarcinoma; Mito C: mitomycin C; DDP: cisplatin; 5-FU: 5-fluorouracil, TS: tegafur suppositories.

The incidence of late, severe complications was significantly related to the factors of higher total dose and brachytherapy dose. In addition to the dose factors, the combined treatment with chemotherapy also significantly increased the incidence of relevant, late complications. Atsunori Yorozu reported that treatment-related esophageal ulcerations or strictures occurred in 18 patients (34%) in a CRT group compared to 12% of patients in an RT group (*p* = 0.013) [17]. The RTOG 92-07 [14] documented treatment-related esophageal fistulas in 12% of the patients. In comparison, no treatment-related esophageal fistulas were reported in several other series of patients who received BT and EBRT without chemotherapy [18-21]. The RTOG 92-07 [14] reported that increased courses of chemotherapy and chemotherapy concurrent with brachytherapy might significantly improve the incidence of late, severe complications. In the present study, the PF regimen significantly increased the rate of severe, late complication compared to the TS regimen (*p* = 0.080). In our experience, CCRT exhibited very low toxicity, which permitted nearly all of the patients to complete EBRT followed, when indicated, by a brachytherapy boost. The reasons that our toxicity scores were lower than those of other reports are probably due to the relatively lower mean dose per fraction of brachytherapy boost and the fact that we utilized extremely individualized treatment schedules that involved subsequent esophagography re-evaluations after every fraction that were used to decide whether the next fraction should be given. In the present study, the incidence of severe, late complications in the CRT group was low. This finding is attributable to several main reasons. First, the chemotherapy regimens were simple, and the majority of patients received the TS alone and lower PF dosage regimens. Second, the concurrent chemotherapy doses were lower than those that are used in normal chemotherapy-alone regimens, which might result in radiotherapy sensitization. Third, we believe that there are at least two factors that made the ^252^Cf-based NBT more effective than the ^192^Ir-based HDR, particularly in the treatment of locally advanced esophageal cancer. The first factor is related to the high-LET nature of fission neutrons, which made them much more effective (compared to the low-LET X-ray) in killing the hypoxic tumor cells in the locally advanced cancers. The second factor is related to the fact that water is an effective neutron attenuator that can be conveniently injected into the source applicator during treatment to reduce the neutron dose to the nearby normal tissue. Because there is a significant difference in the elasticities of normal tissue and tumor tissue, the proper injection of water into the source applicator can effectively push away the nearby normal tissue while still keeping the tumor tissue close to the source. We estimate that 1 cm of water can reduce the neutron dose by approximately 15%.

## Conclusion

In summary, we believe that NBT + EBRT with concurrent chemotherapy is a safe and effective treatment option for patients with thoracic esophageal cancer. Selected patients who were treated with combined chemoradiotherapy and neutron brachytherapy boost achieved better local control. We recommend a regimen of chemotherapy with TS alone.
